# Aligning visual imagery to the operator improves geospatial situation awareness in a single-display 360-degree periscope concept

**DOI:** 10.1186/s41235-025-00646-1

**Published:** 2025-06-23

**Authors:** Jason Bell, Zachary Howard, Stephen Pond, Troy Visser, Madison Fitzgerald, Megan Schmitt, Shayne Loft, Steph Michailovs

**Affiliations:** 1 School of Psychological Science, The University of Western Australia, Perth, WA 6009 Australia; 2 Defence, Science, Technology Group (DSTG), Stirling, WA Australia

**Keywords:** Situational awareness, Visual imagery, Perception

## Abstract

Technological advances mean that it is now possible to represent the entire 360° view of the horizon to a submarine periscope operator simultaneously, in strips on a single display, as opposed to the restricted view offered through a conventional periscope aperture. Initial research showing performance improvements for such panoramic displays is promising. However, that research has yet to consider the importance of alignment between the visual representation of the environment on the periscope display and the operator themselves (i.e. the visual field compatibility principle). Using a simulated periscope operator task, the current study assessed whether the degree of display-operator alignment influences periscope operator geospatial situation awareness (SA). Four increasingly misaligned display configurations and three different operator orientations (relative to simulated Ownship travel) were assessed. Trained novices (*N* = 83) were tasked with judging the position of contacts on their display by pointing a joystick at their “real-world” location to measure geospatial SA. Results revealed a strong influence of display-operator alignment on geospatial SA: an aligned display representing contacts in front of an operator at the top of the display and contacts behind an operator at the bottom of the display, produced better geospatial SA (faster, more accurate responses) than other, less aligned display configurations. Diffusion modelling indicated that greater display alignment improved geospatial SA by both increasing information-processing speed and decreasing the amount of evidence required to make decisions. We conclude that geospatial SA can be facilitated by panoramic designs that maximise the alignment of the display to the external world.

## Introduction

In a conventional submarine environment, a periscope operator works on visual imagery referred to them through an aperture facing a segment of the external environment. A strength of this design is that the periscope operator maintains strong spatial alignment with the external visual environment because their view is tied to the orientation of the periscope apparatus. That is, looking aft or stern from Ownship (i.e. the vessel the operator is positioned within) involves the same physical rotation of the periscope and the operator. On the flip side, in this conventional design the periscope operator has a restricted field of view (limited by the periscope aperture) and therefore must search the external environment in a serial, time-consuming fashion. Technological advances mean that there are now opportunities to represent the entire 360° panoramic view of the horizon to the operator *simultaneously,* on a single display. In our previous work, we found broad performance improvements when using panoramic, compared with conventional periscope designs, with no cost to perceived cognitive workload or perceived system usability (Michailovs et al., 2022, 2024). 

An important but untested consideration in such a panoramic design philosophy is the relationship between the operator and the visual representation of the environment. With a conventional periscope, operators are physically tethered to the imagery. However, even traditional single-bearing view submarine imagery is moving to optronics (i.e. digital images displayed on a static screen; Roberts et al., 2021), rather than periscope-based technology. Kirsch et al. (2013) discuss the concept of ‘embodied cognition’, which posits that our bodily interactions with the environment significantly influence our cognitive abilities. Embodied cognition would suggest that the physical connection of the operator and the imagery in the conventional periscope facilitates cognitive processing, particularly for spatial reasoning. Consistent with this, operator-centric, or egocentric navigational display interfaces (i.e. those that move with the operator to maintain a direct mapping between the operator and environment) lead to faster and more accurate navigational decisions in nautical (Porathe, 2006) and remote-operated vehicle (Cho et al., 2017) contexts, among others. By contrast, the panoramic periscope concept used in our prior studies (Michailovs et al., 2022, 2024) displayed a fixed-position, single screen view (in those studies, fixed to the course of Ownship). The horizon was broken into strips, vertically stacked, to visualise the entire horizon on a single screen, and the strips of the panoramic display were fixed in an arrangement following a clockwise direction to mimic the traditional sweep of the periscope.

The degree of alignment between a visual display and its operator can influence their spatial abilities, including geospatial situation awareness (SA, which in this context we define as the speed and accuracy with which the periscope operator can localise contacts in physical space). The visual field compatibility principle (Worringham & Beringer, 1989) was originally developed in an engineering context to explain the importance of alignment between the movements in the visual field of an operator and their viewed movement at the remote location, i.e. moving a lever left and seeing a remote robotic arm move left, versus right, depending on a camera view. This alignment theory holds for a range of mechanical actions, including rotational movements (Chan & Hoffman, 2016; Hoffman & Chan, 2013), movements of digital items such as mouse cursors (Wigdor et al., 2006), and controlling remotely operated vehicles (Higuchi & Rekimoto, 2013). More broadly, the orientation or alignment of an individual with respect to their environment can impact the efficiency and accuracy with which information can be located in that environment. McCarley and Wickens (2005) attribute a significant proportion of safety-incidents in remote-operated vehicle contexts to mismatched frames of reference, (i.e. pilots misinterpreting spatial information due to inadequate display interfaces). The obvious, potentially catastrophic consequence in a submarine is mislocalizing a contact (vessel), which would be considered a failure to attain geospatial SA (McCarley & Wickens, 2005). 

Spatial judgement is largely dependent on an egocentric frame of reference (Filimon, 2015). For example, Cho et al. (2017) showed that controlling a drone through an egocentric control interface led to faster and better (safer) performance in obstacle avoidance tasks compared with the traditional drone-centric interface. Despite the beneficial aspects of the panoramic periscope concept used in our prior studies (Michailovs et al., 2022, 2024), it clearly reduces the egocentric mapping between the operator and the environment. The operator sits in a fixed location, viewing a static screen that shows a full 360-degree horizon. Translating the location of a vessel or point of interest on a monitor to a real-world location requires mental rotation, from the operator’s point of view to the physical location in three-dimensional space, akin to the drone-centric interfaces discussed above. Accurately and quickly achieving this translation is critical to remaining safe and undetected in the submarine context because having the periscope raised longer than necessary increases the risk of detection. However, mental rotation is cognitively taxing due to its reliance on visual working memory (Wickens et al., 2005, 2010), and results in reduced task accuracy (Conrad et al., 2006; Hyun & Luck, 2007) and slows responses (Provost & Heathcote, 2015).

In fundamental terms, there can be a cognitive conflict in the panoramic display, where concepts displayed on the left of the display may be situated on the right side of the operator, or Ownship. The well-known Simon effect describes how responses in these situations tend to be slower and less accurate, even for very simple decisions (Hommel, 2011; Simon & Small, 1969). Additionally, there is a response cost for making responses in a ‘non-stereotype’ direction inconsistent with expectations (Chan & Hoffmann, 2016; Hoffmann & Chan, 2013). Some simple heuristics, such as forward = up, backwards = down (Wickens et al., 2010), are violated in versions of our panoramic periscope concept, potentially degrading performance further. Wickens et al. (2010) describe the significant impairments that can arise when frames of reference need to be translated in operational settings, particularly when multiple axes of reference need to be translated simultaneously. In addition, there is a wealth of evidence that larger rotations cause further impairments (Gugerty & Brooks, 2004; Gugerty & Rhodes, 2007; Kaltner et al., 2014; Provost & Heathcote, 2015).

Given this literature, the current study sought to assess, in a use-inspired, but controlled, laboratory task, whether the degree of display-operator alignment influences periscope operator geospatial SA, and importantly, whether we could improve that geospatial SA while preserving the panoramic concept. With our operational definition of geospatial SA as “quickly and accurately localising contacts”, we developed a paradigm where participants used a joystick to rapidly indicate where a series of contacts were in physical space, in relation to their viewpoint. In addition to analysing raw Response Time (RT) and accuracy, we fit an Evidence Accumulation Model (EAM) known as the Racing Diffusion Model (RDM; Tillman et al., 2020) to the combined data (RT + accuracy). EAMs provide a way to distil observed behavioural responses into latent theoretical constructs that describe how evidence for a decision is accumulated and made. They have seen widespread application to simple decision tasks in cognitive psychology and neuroscience (Forstmann et al., 2016; Heathcote & Matzke, 2022; Ratcliff & van Dongen, 2011) and more recently have been used to gain insight into the cognitive processes that underlie observed behaviour in applied domains, such as air-traffic control, driving, forensic and medical image discrimination, and maritime surveillance (for a review, see Boag et al., 2023). At the most basic level, EAMs have three key parameters: (1) Drift Rate [v], (2) Threshold/Boundary Separation [a], and (3) Non-decision time [t0] (described further in the Method). By observing how these parameters change across experimental manipulations, we can infer what psychological mechanisms underpin any observed variations in geospatial SA. For example, faster RTs associated with an increased drift rate would suggest that a given display configuration improved the quality of information that was available to be processed about contact location. By comparison, faster RTs associated with a lower non-decision time would suggest that a given display configuration facilitated motor responses. It is also possible that a given display configuration could influence response caution (decision thresholds—the amount of evidence required to decide).

### Display configuration

In the submarine periscope domain, we identified three distinct dimensions of alignment: (1) the degree to which the display configuration aligns with the surrounding environment, (2) the operator’s positional orientation relative to the direction of travel of Ownship, and (3) the interaction between display content and operator orientation (i.e. whether the segment of environment shown at the top of an operator’s display is fixed or tailored to their orientation within Ownship).

### Display alignment

In the Michailovs et al. and’s (2022, 2024) initial studies on the panoramic periscope display concept, the panoramic strips were arranged in an order that mimicked an operator sweeping the horizon in a clockwise fashion with an optical path periscope (see Fig. 5, top-left). While this layout maintains continuity between strip edges, it disrupts the spatial configuration of imagery [e.g. contacts appearing on the left side of the display may actually be on the starboard (right) side of Ownship, see the blurring of red (left) and green (right) across the display]. In addition, simple heuristics like forward = up, backwards = down may be violated, particularly as the strip representing the environment directly behind Ownship is one of the middle strips. To examine whether the layout of strips in a panoramic concept could optimise geospatial SA, we compared several increasingly more “spatially congruent” displays, as well as an intentionally disordered display. These varyingly aligned configurations are presented in Fig. 5 and further described in the Methods.

### Operator orientation

With a conventional periscope design, the orientation of an operator is tethered to that of the periscope mast: meaning that a visible contact is always in front of the operator, and embodied cognition may assist with spatial awareness (Kirsch et al., 2013). In contrast, for a fixed digital display such as the panoramic design considered by Michailovs et al., (2022, 2024), there is no need or capacity to physically turn to face a contact when viewing it. This raises the question of whether decoupling the operator orientation from Ownship direction comes at a cost to geospatial SA and raises work design questions such as where the operator should be positioned in the control room. The counterpoint is that due to the 360-degree nature of periscope viewing, no one operator orientation has an inherently higher degree of mental rotation demands to another (i.e. it is possible that the static nature itself, rather than any specific orientation, is the critical distinction from traditional periscope masts). To provide a broad assessment of these questions, we designed three alternative participant orientations, with respect to the simulated orientation and direction of travel of Ownship. Participants could complete their tasks while facing forward (FWD), starboard (STBD), or aft (AFT) with respect to the orientation of Ownship*.*

### Display alignment by operator orientation

The most aligned visual configuration of the panoramic display is not absolute, but rather, depends on the orientation of the operator. For instance, if the operator was facing forward during northwards travel, the topmost strip would span a 90° centred on due north/0°. But for a starboard facing operator in this scenario, alignment means that the topmost strip needs to shift 90° to represent the 90° arc centred on east/90° (i.e. the starboard side which is in front of a starboard operator, to match the heuristic forward = up). Thus, to create analogous alignment manipulations for starboard and aft operator orientations, each of the display configurations described above was replicated but rotated through 90° or 180°, respectively. Where the panorama optimisation matches the operator’s orientation (e.g. starboard environment in the top strip for starboard facing operators), we term this as an *aligned* condition. In an *aligned* configuration, a contact’s external location is reliably cued from its display location. Where the optimisation does not match the operator’s orientation (e.g. forward environment in the top strip for starboard facing operators), the condition is *misaligned*. Here the location of a contact on the display does not reliably cue its external location. Appendix contains examples of how optimisation impacts the display for different operator facing directions.

### Current study

In summary, new technology offers the opportunity to present more of the external environment to periscope operators via a panoramic 360° display. While benefits to this design have been reported (Michailovs et al., 2022, 2024), the importance of maximising the alignment of display information for the operator’s view has not been investigated. Therefore, the aim of the current study was to assess the influence of display configuration and operator orientation on operator geospatial SA. To measure geospatial SA, we designed a speeded response task where participants were required to rapidly estimate the physical location in the world of a contact on their display by pointing a directional joystick. All contacts were programmed to be readily visible on the display so that performance was minimally influenced by visual search ability. Given the likely influence of general spatial ability in performing our contact localisation task, we assessed individual differences in spatial working memory.

### Hypotheses

Display alignment: Assuming the visual compatibility field principle holds in a simulated periscope operator role, we hypothesised (1) that participants would have best geospatial SA in the Spatial condition, followed by the Partitioned, Clockwise, and then Disordered configurations (Fig. 5).

Operator Orientation: Given that the amount of mental rotation required by each display configuration was constant across all operator orientations, we hypothesized (2) that operator orientation [i.e. forward (FWD), starboard (STBD), aft (AFT)] would not influence geospatial SA.

Display-operator alignment: Given our conjecture that the alignment of the display to the operator’s orientation is fundamental to geospatial SA, we hypothesised (3) that participants would have better geospatial SA in the aligned condition than the misaligned conditions, irrespective of display configuration. This hypothesis would be supported by an interaction effect between Display alignment and Operator Orientation.

Diffusion modelling: We treat the modelling as exploratory. Briefly, we would expect to see increased Drift Rate (with increased alignment) if a spatial aligned display configuration improve the *quality of information* about a contacts’ location, reduced Threshold (with increased alignment) if a spatially aligned display increased a participant’s *confidence* (reduced response caution) around the evidence about a contacts’ location, and reduced non-decision time (with increased alignment) if a spatially aligned display configuration speeded response execution after the contact location had been determined (e.g. minimising Frame of Reference transformation in the execution phase).

## Method

### Participants

Eighty-three participants took part in the experiment in exchange for course credit or $30 AUD. This research complied with the National Statement of Ethical Conduct in Human Research (code of conduct) and was approved by the Human Research Ethics Office at the University of Western Australia. Informed consent was obtained from each participant. Participants were sampled from the University research pool primarily consisting of undergraduate psychology students (with no experience in submarines), but specific demographic details (age, gender) were not collected as we had no theoretical need to do so (see, e.g. Trafimow et al., 2023).

### Materials

**Fig. 1:**
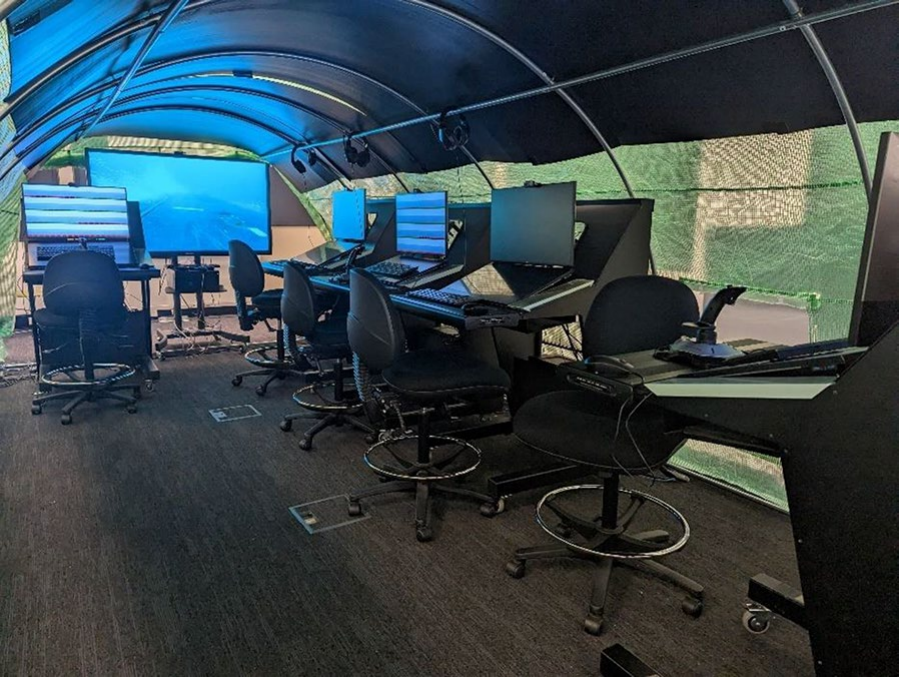
An illustration of the canopy constructed to simulate a sense of Ownship orientation relative to ‘facing forward’ and ‘facing starboard’. Note that during the experiment all monitors were blank, except the 65-inch display front looped display, and the top display of consoles being used by participants in their respective orientation

The experiment was conducted inside a 6-m-long, 3-m-wide, 2-m high-framed canopy that invoked the tunnel shape of a submarine (Fig. 1). Three computer consoles were set up at different orientations (forward, starboard, and aft) with additional unused consoles placed along the starboard side (the unused consoles were part of the lab but not used for the present study—the middle console was used for the starboard-facing condition). Up to three participants (one per orientation) participated at a time, depending on sign ups. To facilitate a sense of Ownship orientation, a 65-inch display was mounted at the forward end of the tunnel. This played a looped video showing an external view of front of a submerged submarine in motion, with streams of oncoming bubbles cuing the direction of travel. The display also played a recording of submarine engine noise to assist with immersion and to minimise any extraneous sounds.

**Fig. 2:**
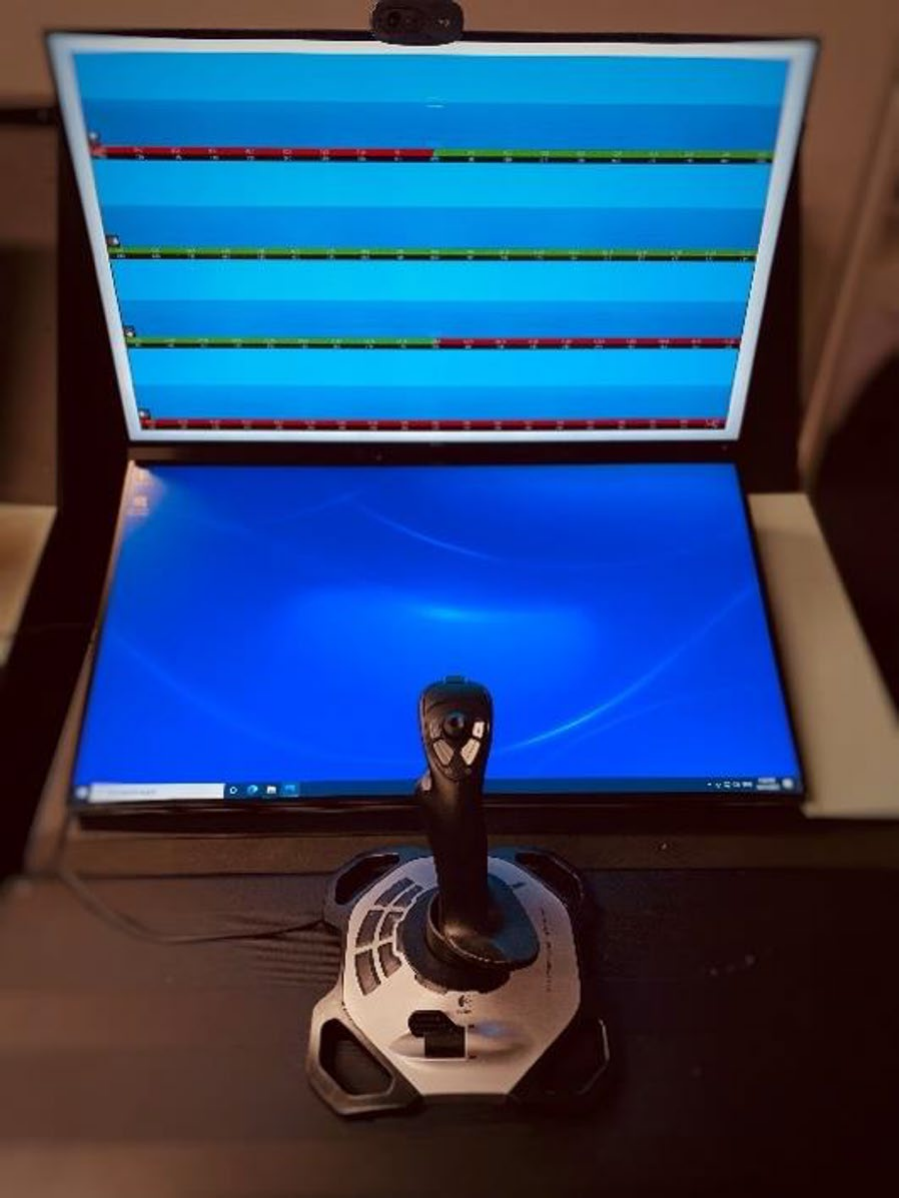
Panoramic display shown in top display, with response joystick in front. The bottom display was left blank during the tasks. The clockwise configuration is shown

The experiment was programmed and controlled using PsychoPy v2022.1.3 (Peirce et al., 2019), which displayed on a 27-inch LCD monitor on the top screen of the console (Fig. 2). The bottom screen of each console was blank. Directional responses were made via Logitech Extreme 3D Pro joystick. Responses were recorded if the joystick entered one of eight zones of 30° each, and the joystick was pushed a distance equivalent to 95% of the way towards the limit of the range of motion. The eight zones corresponded to cardinal (front, right, etc.) and oblique (front-left quadrant, etc.) axes.

### Spatial ability

Digitised versions of two standardised tests of spatial ability were included. Spatial working memory storage has been shown to be important in the visual search process (Oh & Kim, 2004) and the ability to mentally manipulate or rotate objects (Hyun & Luck, 2007). Thus, assessing spatial ability was important to control for individual differences across participants.

**Fig. 3:**
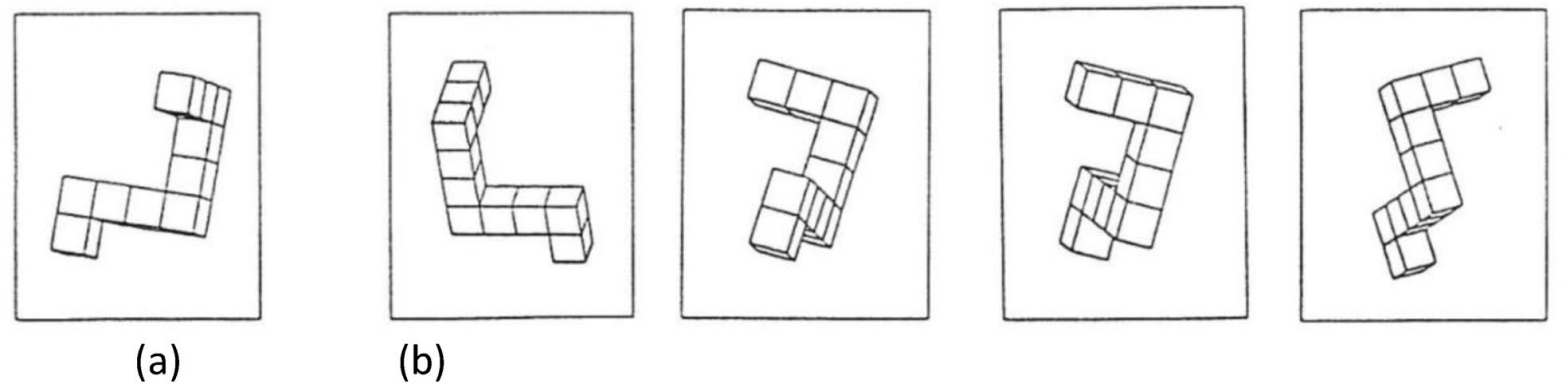
Mental rotations test (MRT). Participant must match the first object (**a**) with two from a set of four (**b**). Examples based on AUTOCAD drawings of Vanderberg and Kuse (1978) stimuli kindly provided by Michael Peters, University of Guelph

In the Mental Rotations Test (MRT; Vendenberg & Kuse, 1978), as adapted by Peters et al (1995), 2D representation of a 3D object must be matched with the same object drawn from a different perspective from among four options. The test consisted of 24 items and had a time limit of six minutes (see Fig. 3).

**Fig. 4:**
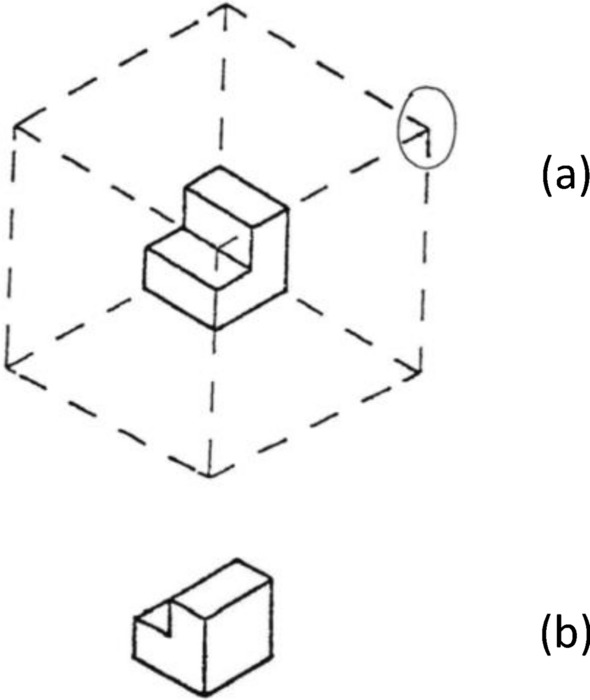
Visualisation Viewpoints Test (VVP). Participant must identify (circle) the corner of a cube (**a**) from which the object (**b**) was viewed. Answer is shown

In Guay’s Visualisation Viewpoints Test (VVP; Guay & MacDaniels, 1976), as adapted by Eliot and Smith (1983), a 3D object is drawn once as though within a transparent cube, and once from another perspective. Participants must identify which corner of the cube the second image is seen from (i.e. they must imagine viewing the object from different perspectives). The test consisted of 24 items and had a time limit of eight minutes (Fig. 4).

### Display concepts and stimuli

**Fig. 5:**
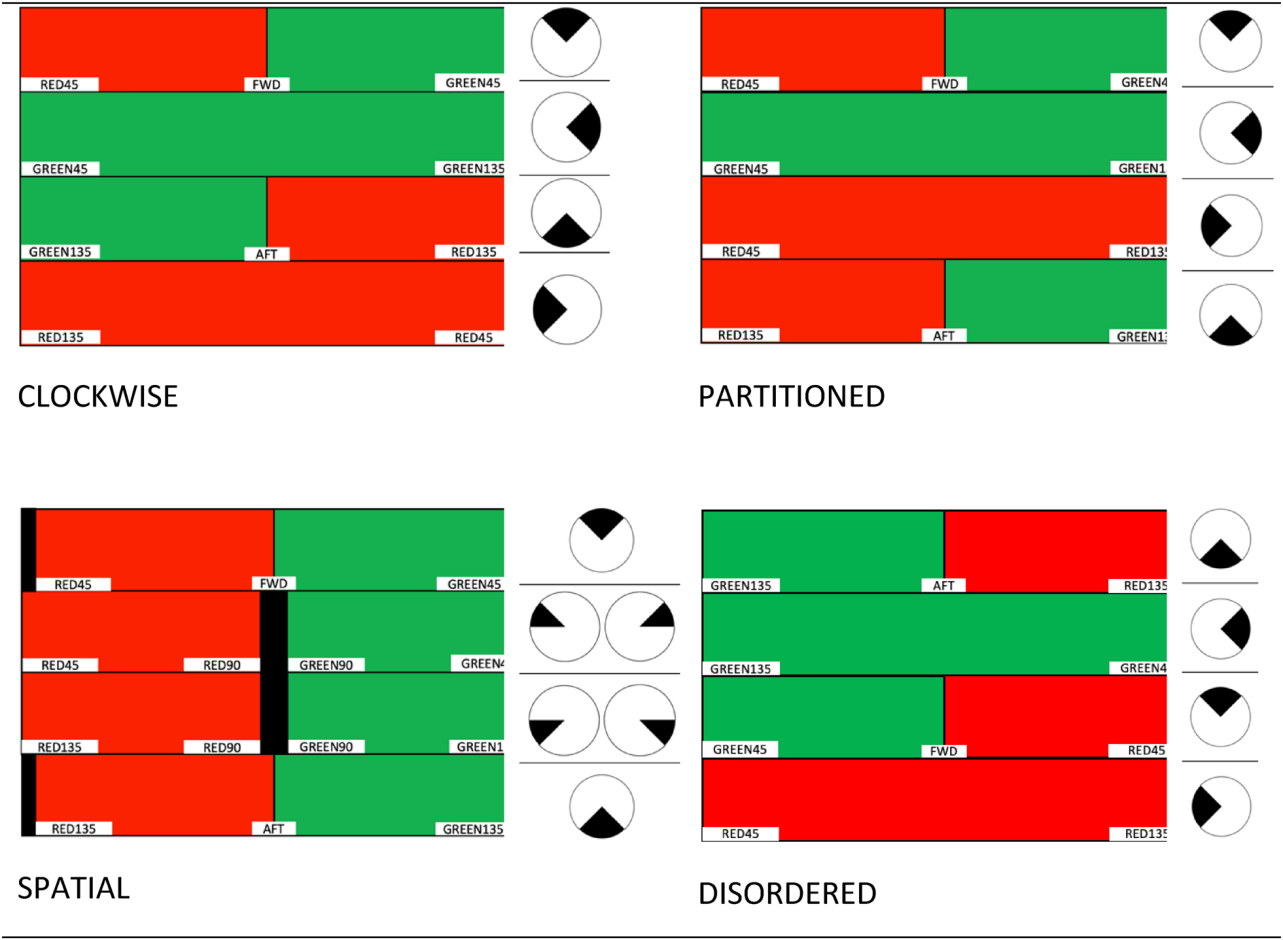
Examples of the four display configurations of a 360-degree panoramic periscope display tested. In each strip, red represents port (left) side of Ownship, while green represents starboard (right). The filled arc in the circle/s adjacent to each strip illustrates the segment of horizon displayed in that strip

Four periscope display configuration concepts were developed, each showing a panoramic view of the full 360° horizon across four vertically arranged strips. See below for a description of the four differing configurations: Clockwise, Partitioned, Spatial, and Disordered (Fig. 5). For each configuration, three separate versions were developed, to preserve the alignment with the external environment in each operator orientation, such that each is optimised for a particular orientation. See the Appendix for the full set of configurations and optimisations.

To investigate this display-operator alignment issue more systematically, below we describe a series of display configurations that are constant in scope (360°) but vary in the order of imagery shown on each horizontal strip (see illustrations in Fig. 5). In order, the conditions described below decrease in alignment/compatibility with the operator. Descriptions are for a forward-facing operator viewing a single display.

*Spatial condition *(Fig. 5*, bottom left*)*:* A maximally (4/4 strips) aligned single visual display for a forward-facing periscope operator means that contacts in front of Ownship are in the top strip of the display, those aft/behind are in the bottom strip, while contacts on the portside (to the left of Ownship) are on the left side of the display, and contacts on the starboard side (to the right of Ownship) are on the right side of the display.

*Partitioned condition *(Fig. 5*, top right*)*:* The second most aligned (2/4) configuration is one where the top strip presents contacts in front of the operator and the bottom strip presents those behind. The second (starboard) and third (port) strips now also present continuous 90° segments. These strips are only partially aligned in that, a contact on the left of the second (starboard) strip is not aligned with its physical position to the right of Ownship/operator, but a contact on the right of the same strip is broadly aligned.

*Clockwise condition *(Fig. 5*, top left*): The third most aligned configuration is the clockwise layout, which maintains alignment for the top strip only (1/4). As explained above, the second (starboard) and fourth (port) strips are only partially aligned, and adding to the misalignment, now aft is represented in the 3rd strip, not the bottom. This condition is an effective replication of the Michailovs et al., (2022, 2024) study conditions, barring the reduction from five strips there, to four here.

*Disordered condition:* The fourth configuration arranged the strips to maximally misalign (0/4) with the external environment. The top strip shows the aft section, and both the aft and forward strips have port to the right and starboard to the left, i.e. are reversed. This condition provides an important baseline comparison to the other configurations, allowing us to establish whether any degree of alignment to the environment improves operator geospatial SA.

As our main measure of geospatial SA, participants were tasked with judging which direction a contact was located in relation to their current facing orientation, by “pointing” to that contact using the joystick. For example, in Fig. 2 where a contact was shown at the Forward location on the display (i.e. in front of Ownship), a participant facing forward would point the joystick forward, whereas a participant facing starboard would point the joystick left, and a participant facing aft would point down (behind them). A target contact model could be presented at any of 16 locations on the panorama display, representing eight distinct responses at 45° intervals (the diagonal locations appeared on multiple strips). Each target subtended a minimum visual angle of 1.12° when viewed from approximately 60 cm; i.e. they were readily visible to participants.

### Design

The experiment followed a mixed measures design with Display Configuration (Clockwise|Partitioned|Spatial|Disordered) and Display Optimisation (for FWD|for STBD|for AFT) as between-subjects factors, and Operator Orientation (facing FWD|facing STBD|facing AFT) as a within-subjects factor. Thus, each participant was tested in all three facing orientations but only experienced a display-optimisation pair (i.e. aligned to the direction they were facing) in one of the three orientations. (In fact, the participant’s interface was identical throughout all sessions to minimise learning challenges and better capture the influence of the orientation change.) Order of orientation was counterbalanced such that for each display-optimisation pair, one participant completed the experiment in each of the six possible orders.

### Procedure

Up to three participants completed the experiment simultaneously. Participants were seated at their first console and watched a 5-min training video explaining the task, the orientation of the submarine, and the concepts of port, starboard, and relative bearing. The experimenter then asked participants to point to various relative bearings in the room with their hands to gauge whether they had understood the training, before continuing. Participants then completed the MRT and VVP spatial ability tests using the mouse to enter responses. Finally, participants were introduced to the joystick and the eight response directions. Participants viewed the panorama, once on its own and once with arrows at each of the 16 possible target locations, representing the correct answer for a target at that location, given their current facing orientation. Practice began with 16 direction trials, in which participants used the joystick to respond to a relative bearing (e.g. “Where is Red 45?”). They then completed 32 practice trials of the task proper. Both practice tasks included feedback in the form of an arrow representing the correct response direction.

The primary experimental task consisted of 240 trials involving a single target contact. Trials ended when the participant responded, or after 5 s, and were separated by an inter-trial interval of 1 s in which the panorama remained visible. Response time and continuous error (from the contacts exact location) were recorded, but accuracy was re-coded to “within correct 45-degree segment” for analysis. Two self-timed breaks occurred at intervals of 80 trials.

Individual participants remained seated until all participants had finished the session. They were then moved to the next orientation position to complete the next session.

## Results

### Descriptive statistics

We excluded any participant who failed to achieve at least 50% accuracy in any of the three Orientation conditions. The task was relatively simple (mean accuracy of remaining participants was 94.01%), and as such low scores would suggest a failure to engage. This resulted in the removal of seven participants, leaving a sample of *n* = 76 remaining for analysis. All seven excluded participants showed higher (> 50%) accuracy in at least one of the three sessions, suggesting these participants failed to adapt to the change in orientation. For all RT analyses, we excluded RTs less than 100 ms (which could only be achieved by anticipating target onset), or more than 5000 ms (the timeout for the trial).

Participants showed large variability in spatial ability scores, with scores ranging from 2 to 22 correct, out of a possible 24 on the MRT, and from 2 to 23 correct out of a possible 24 on the VVP. The mean score was 11.30 (SD = 4.27) on the MRT and 13.22 (SD = 5.34) on the VVP. The two scales were moderately correlated (*r* = 0.46, BF_10_ = 704.04), suggesting they captured related but distinct aspects of spatial ability. At the group level, there was no statistical difference in either MRT (Posterior BF_null_ = 15.066) or VVP (BF_null_ = 16.92) score when split by assigned configuration or display-optimisation, suggesting that participants’ ability levels were well distributed across the experimental conditions and thus were unlikely to account for performance differences across conditions. This is important, because as can be seen in Table 1, there was strong evidence that higher spatial ability led to faster geospatial SA decisions. Spatial ability was unrelated to baseline RT, showing that the observed effects in Table 1 are related to the spatial reasoning component of the task and not to differences in baseline response speed.

**Table 1:** Correlations between experimental performance and spatial memory scores

	MRT	VVP	RT (Experiment)	Accuracy	RT (Baseline)
MRT	–				
VVP	**0.458**	–			
RT (Experiment)	− **0.413**	− **0.394**	–		
Accuracy	0.204	0.267	− **0.394**	–	
RT (Baseline)	− 0.240	− 0.241	**0.465**	-0.135	–

NB. Bolded correlations show strong (BF > 10) evidence

### Response time

Given the high overall accuracy (*M* = 94.01%) on the localisation task, we expected the primary differentiator of performance to be the time taken to make a decision about a contact’s spatial location (RT). We analysed RTs for correct responses only. The mean RT by condition data is reported in the Appendix, including all inferential analysis. A three-way Bayesian ANOVA on RT, with a subject-level random effect (intercept), showed that all main effects and interactions were included in the preferred model, indicating performance depended on the relationship between the display configuration, operator orientation, and display optimisation. However, when unpacking these statistics, it was clear that the dominant effect was a three-way interaction in which operator orientation and the direction of console optimisation were closely related, and this “alignment” effect was stronger for some configurations than others (i.e. the absolute orientation was less important than how orientation and console optimisation were combined). Operator Orientation credibly impacted RT, such that facing forward led to faster responses (BF_10_ > 1000), but only when the display was console was optimised for the orientation (BF_01_ > 1000 for misaligned conditions only). Likewise, Partitioned then Spatial configurations led to faster responses, but only when the Orientation and Optimisation matched (BF_10_ > 1000; when misaligned the effect remained credible but Partitioned was faster than Spatial). To highlight this relationship, we reduced the experimental factors to Configuration x *Aligned*, where the configuration is operationalised as aligned when the operator’s orientation matches the optimisation of the display.

**Fig. 6:**
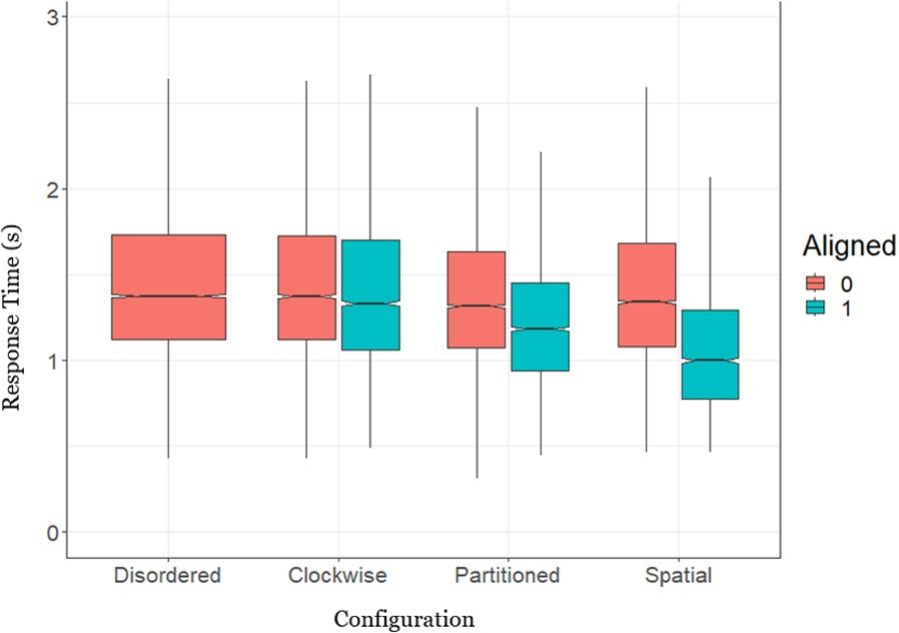
Response times as a function of display configuration and alignment. Typically, in a notched box-plot any boxes whose notches do not overlap can be considered statistically different (the notched area represents the 95% credible interval around the median RT). The corresponding GLMM results are presented in Table 3

Figure 6 presents mean RTs for each display configuration when it was aligned (teal) or misaligned (red) for that operator’s orientation (and see Table 2). The Disordered configuration cannot be aligned (by design) and thus has no aligned counterpart. To test the effect of Configuration and Alignment (of display to operator orientation), we performed a Bayesian Generalized Linear Mixed-Effects (GLMM) model with an inverse Gaussian distribution and an identity link function, which better reflects the skewed nature of RTs and improves power over a traditional ANOVA (see, e.g. Lo & Andrews, 2015). Coefficients are in the same units as the dependent variable, in this case RT in seconds. The model output is included in Table 3. There was a strong effect of Alignment, and an interaction between Configuration and Alignment. There was evidence against the effect of Configuration on its own. As can be seen in Fig. 6, Alignment produced faster RTs in the Partitioned and Spatial configuration conditions but had no impact on RTs for the Clockwise condition. As hypothesized, the performance advantage of alignment is strongest in the Spatial configuration, such that an aligned Spatial configuration is the fastest of all conditions tested, but a misaligned spatial configuration is as slow as a disordered one. Noting that median RT across conditions was 1.33 s, practical effect sizes here are large, with the fastest (median response time) condition (Spatial/Aligned) being over 0.40 s faster than the slowest condition (Clockwise/Misaligned).

**Table 2:** Median RT, mean accuracy, and 95% CIs by configuration and aligned

Configuration	Misaligned	Misaligned	Aligned	Aligned
	RT (95% CI)	Accuracy (95% CI)	RT (95% CI)	Accuracy (95% CI)
Disordered	1.396s (1.385, 1.406)	0.929 (0.924, 0.933)	NA	NA
Clockwise	1.407s (1.392, 1.422)	0.915 (0.910, 0.921)	1.360s (1.339, 1.381)	0.942 (0.936, 0.949)
Partitioned	1.333s (1.320, 1.348)	0.941 (0.937, 0.946)	1.187s (1.170, 1.203)	0.962 (0.957, 0.967)
Spatial	1.355s (1.342, 1.369)	0.956 (0.951, 0.960)	1.001s (0.987, 1.015)	0.969 (0.964, 0.974)

**Table 3:** Results of GLMM on RT and accuracy

Parameter	Coefficient (RT)	BF_10_ (RT)	Odds ratio (Acc)	BF_10_ (Acc)
Intercept	1.54	> 1000	N/A	> 1000
Clockwise*	0.07	0.043	0.92	0.096
Partitioned*	− 0.08	0.051	1.30	0.141
Spatial*	− 0.06	0.041	1.51	0.359
Aligned^	− 0.38	> 1000	1.48	25
Clockwise:Aligned†	0.32	> 1000	1.04	0.052
Partitioned:Aligned†	0.24	> 1000	`.09	0.064

*Reference = Disordered; ^Reference = Misaligned; ^†^Reference = Spatial:Aligned

### Accuracy

**Fig. 7:**
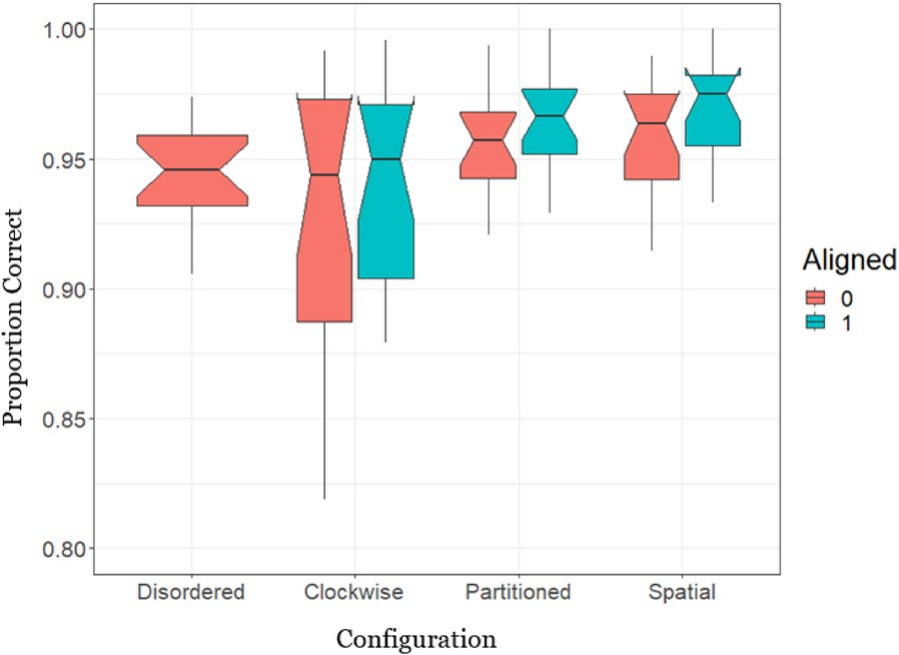
Proportion of correct responses by configuration and alignment. Typically, in a notched box-plot any boxes whose notches do not overlap can be considered statistically different (the notched area represents the 95% credible interval around the median proportion correct)

Although accuracy on our geospatial SA task was near-ceiling levels (Table 2), we analysed the accuracy data to ensure it did not undermine our interpretation of the above RT results (e.g. speed accuracy trade-offs). We performed a Bayesian GLMM with a binomial distribution on the likelihood of responding correctly on each trial. (The continuous error response was coded as within/without the correct 45-degree region for all accuracy analyses.) The only statistically credible effect on accuracy was alignment, where an aligned configuration was more accurate than a misaligned one (Table 3). Although there was no statistical evidence for an interaction (likely due to the near-ceiling performance), visual inspection of Fig. 7 suggests that Alignment facilitated accuracy in the Partitioned and Spatial Configurations specifically, which accords with the RT results.

## Racing diffusion modelling

**Fig. 8:**
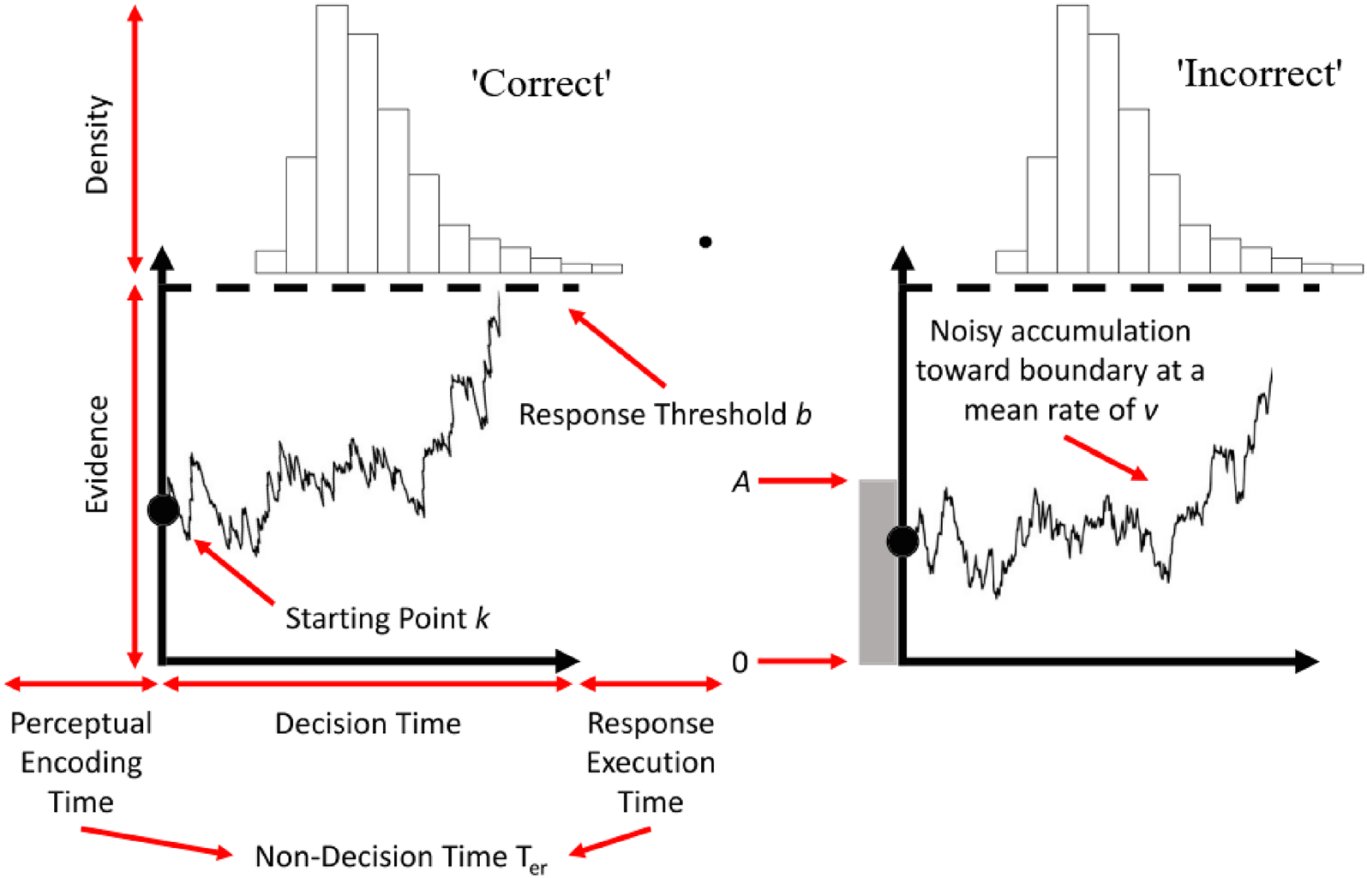
Illustration of a typical racing diffusion model process. Evidence separately accumulates for each of *n* alternatives (in this figure there are two, representing a left or right response). The height of the decision bounds describes the participant’s level of response caution (decision threshold—how much evidence is needed before a decision will be made, thus higher numbers reflect *slower* responding). The rate (mean slope) of evidence accumulation describes how much information per unit of time can be extracted from the stimuli or task environment, with higher numbers reflecting *faster* processing and responding. The perceptual encoding and response execution phases combine to form the non-decision time—that is, time that forms part of the observed RT but is not related to the accumulation of evidence (thus higher values reflect *slower* responding). In a race model, whichever accumulator crosses a bound first determines the winner. For our task, there are eight total accumulators representing the eight possible responses. Figure reproduced from Fig. 2 of Tillman et al (2020)

The above results demonstrate that the Configuration, and particularly the Alignment of a periscope display configuration to the operators’ orientation, influence the speed and accuracy with which they localized contacts on the horizon. However, to gain insight into potential latent cognitive mechanisms underlying these differences, we combined the RT and Accuracy results and modelled them using the Racing Diffusion Model (RDM) approach (Fig. 8; Tillman et al., 2020). We use a race model because our design extends beyond the two choices captured by the standard Diffusion model. Using a hierarchical Bayesian framework through the EMC2 package (Stevenson et al., 2024), we estimated drift rate, threshold and non-decision time parameters for each Configuration [between-subjects] x Aligned [within-subjects] combination, resulting in four separate models (each condition was fit separately, with free parameters for drift, threshold and non-decision per alignment in each condition except for Disordered). We fit a variety of reduced models; however, the overwhelming evidence was in favour of the full model using all selection criteria so we do not report on the other models. To reduce complexity in our eight-accumulator model (reflecting the eight unique responses), we constrained the seven erroneous accumulators to have the same mean drift rate, which assumes there was no inherent bias towards one response or other, and therefore, errors were assumed to primarily result from motor error. (The error drift was a single free parameter that did not vary with Alignment.) Given the high accuracy rates we felt this was appropriate, and there would not be sufficient error data to estimate response-option bias. The height of the start rate variability, and the coefficient of drift, were fixed (not estimated) to allow direct comparison of parameter estimates between models. Model fits are provided in the Appendix, and the models captured the trend results between conditions well.

**Fig. 9:**
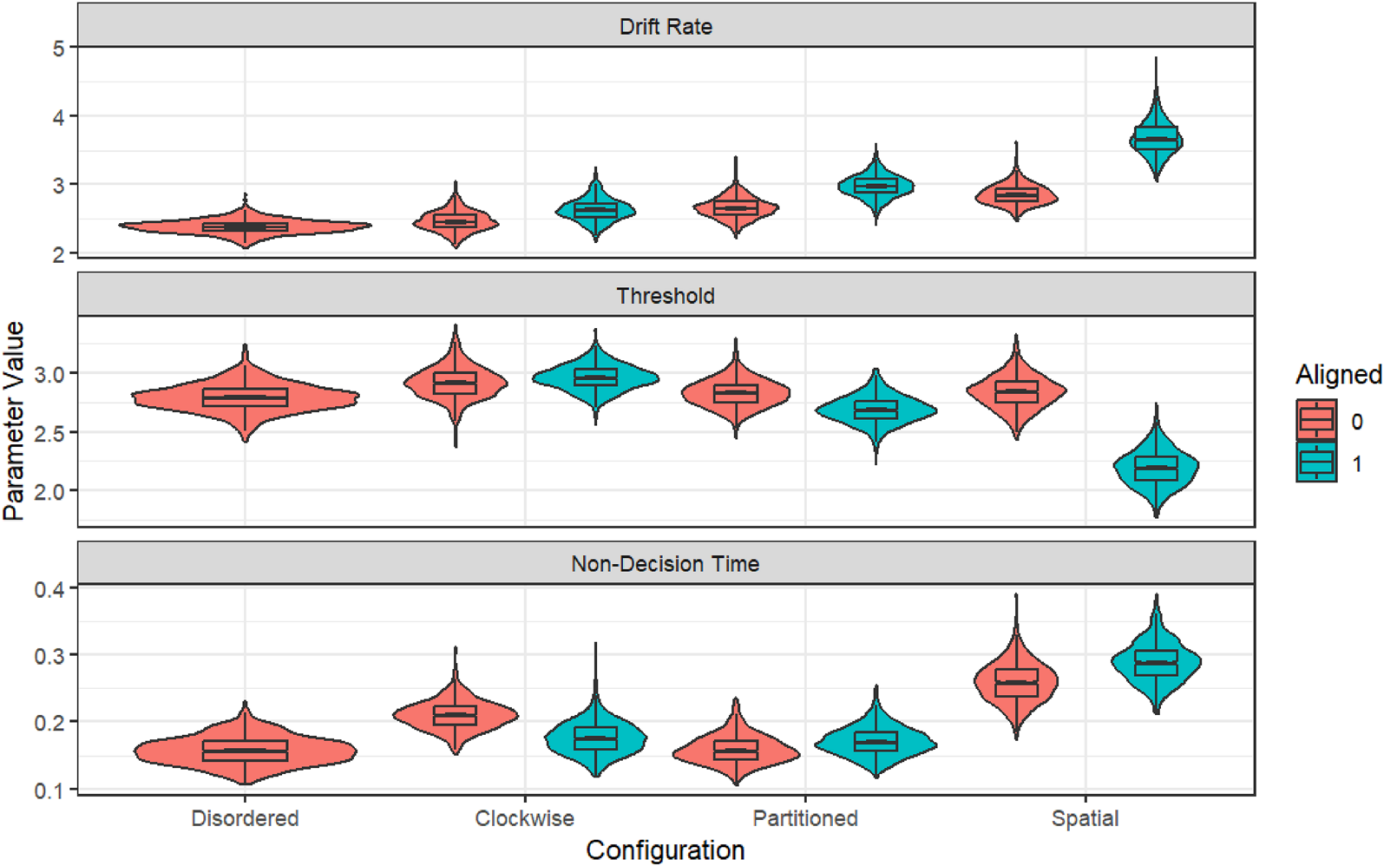
Group-level posterior parameter estimates from the racing diffusion model by configuration and alignment. Typically, in a notched box-plot any boxes whose notches do not overlap can be considered statistically different (the notched area represents the 95% credible interval around the median parameter estimate, here they are very small due to the large number of posterior samples and good model convergence). The overlayed violin plots show the full distribution of the posterior parameter estimates, primarily included to show the model converged to a precise estimate in all cases

The results of the RDM are presented in Fig. 9. We observed statistically credible effects on all parameters. Drift rate was higher for Aligned than Misaligned configurations (all BF_10_ > 1000), but the effect was more pronounced for Partitioned, and particularly Spatial, configurations. Similar results were observed for threshold, which was lower in Aligned than Misaligned (BF_10_ > 1000), but more so for Partitioned and especially Spatial configurations. Significant, but small effects of Alignment were observed for non-decision time. Visual inspection suggests that non-decision time was higher in Spatial than for other configurations; however, this was almost certainly a compensatory result to shift the distribution right given the combination of higher drift rate and lower threshold in that condition. Overall, the modelling results show that as spatial alignment of a display increases, alignment to the environment becomes more critical, both for increasing quality of information uptake from the environment (drift) and for reducing caution on response execution (threshold).

## Discussion

Digital technology has led to the removal of physical periscope apertures from some modern submarines, freeing up much-needed space in the control room. Development of display concepts that can represent the entire 360-degree horizon simultaneously on a single console have further cemented static-screen-based imagery interactions (Michailovs et al., 2022, 2024). However, while these panoramic displays can increase available information, it remains unclear how reducing the physical tethering between operator and environment, a key aspect of embodied cognition (Kirsch et al., 2013), could impact operator performance and particularly geospatial SA. The layout of the panorama on the display (relative to the physical environment) and the operator’s orientation (relative to Ownship) specifically could impact operator visuo-spatial awareness, and it may be possible to optimise these static displays to enhance operator geospatial SA. We assessed the geospatial SA of individuals (defined here as an operator’s speed and accuracy at translating a contact location on a panoramic display to its direction in physical space) under a range of display configurations and operator orientations, to examine the impact of configuration and orientation.

Hypothesis 1 predicted that the Spatial and Partitioned configurations would yield better geospatial SA compared to the Clockwise and Disordered configurations, regardless of orientation or optimisation. This hypothesis was partially supported. Greater geospatial SA for Spatial and Partitioned configurations was found but was limited to conditions in which there was display configuration-operator alignment.

Hypothesis 2 predicted no effect of operator orientation separate from alignment, which was also only partially supported by the findings in that although there was a slight advantage for facing forwards, this only occurred when the display was optimised for that orientation. This suggests that the absolute direction that an operator faces has less effect on geospatial SA than the optimisation of the console, but there may be an inherent “facing forwards” advantage.

Hypothesis 3 predicted greater geospatial SA in Aligned conditions, which was strongly supported by the RT data. Improvement with aligned display panels occurred in the Spatial and Partitioned configurations, with greater response accuracy as well as faster RTs. Together these findings suggest that configuration design and alignment are interdependent: alignment yields geospatial SA benefits when a configuration is designed to facilitate geospatial SA. In fact, the spatial configuration led to the equal *worst* performance when misaligned, highlighting how increased spatial mapping requires a corresponding shift in operator positioning.

The greatest benefits of alignment on geospatial SA were found for the Spatial configuration, with benefits also observed in the Partitioned configuration. Importantly, the RDM results extend the mean RT analyses by providing a mechanistic explanation for *why* responses were faster. The RDM showed that the faster responses were driven by improved information-processing speed in the Spatial condition, followed by the Partitioned condition, compared with the other conditions. In the Spatial configuration, every strip on the display reliably cued a contact’s external location from its display location. In the Partitioned configuration, alignment was not congruent on every strip but, rather, was limited to the top and bottom strips. Therefore, we can conclude that the degree to which the environment and periscope display align fundamentally improves geospatial SA by improving the quality of information about contact locations (thereby increasing information-processing speed) and by decreasing response caution. Although visual information was identical across configurations, differing only in how it was arranged on displays, the RDM revealed that increased alignment improved both the *quality* of information uptake (via increased drift rate) and reduced the *cost* of spatial transformation (via lowered decision thresholds—less response caution). Layout changes did not simply remove a processing step—they enhanced the cognitive encoding of spatial information itself. The finding that alignment benefits were reduced for the Partitioned configuration is consistent with the overall degree of alignment being a key factor for geospatial SA.

Notably, the lack of any significant alignment benefit in the Clockwise conditions further supports this interpretation. In the Clockwise configuration, only the top strip aligned with the environment. This condition was included as it matched the display configuration used by Michailovs et al., (2022, 2024). The Clockwise configuration can be read from left to right and top to bottom to mimic a full sweep of the horizon conducted by an optical path periscope and arguably represents a logical default design. Our findings indicate that this layout may not be optimal for supporting operator geospatial SA—in fact it performed about as poorly as an entirely disordered baseline display.

Interestingly, there were only very small differences observed between the Misaligned conditions. The Disordered configuration was designed so that there was no logical relationship between the strips of the display, yet it performed no worse than the Misaligned version of the other display configurations, even though each of those maintained an internal spatial relationship between strips. It was assumed that the relationship between spatial locations and display positions would be informative even in a misaligned orientation, despite that it would require a fixed mental rotation of 90° or 180°. However, the strips’ relationship to each other in and of itself provided no benefit to geospatial SA. Both display strips that were misaligned but maintained a spatial relationship and display strips that were entirely disordered required mental transformation to indicate the contact location, thus impairing overall geospatial SA.

This interpretation of all Misaligned conditions requiring equivalent mental rotation was bolstered by the fact that decision threshold—which reflects the amount of evidence required before executing a decision—yielded large differences between layouts (particularly for the Partitioned and Spatial configurations). Thresholds were equally high (resulting in slower responses) in the more spatially arranged strips when they were misaligned, compared with the other less spatially arranged strips, suggesting the response conflicts are exacerbated as the presumed relationship between the display and environment becomes stronger, but greater benefits can be had by optimising the alignment. Although some research has linked non-decision times to spatial rotations (particularly in same/different paradigms where rotations likely happen *before* decisions are made; Feldman & Huang-Pollock, 2021), other research has found predominantly response threshold effects (e.g. Provost & Heathcote, 2015). In particular, increased thresholds have been linked to multi-tasking (specifically the addition of tasks; Howard et al., 2020). 

In our paradigm, it seems likely that mentally rotating between display and real-world locations constitutes an additional processing stage that is reduced or removed with spatially aligned displays (response threshold effect). The progressive decrease in threshold from, e.g. Disordered to Partitioned (aligned) to Spatial (aligned) suggests a decrease in the number of axes to be translated—i.e. the higher the degree of alignment, the lower the proportion of transformations that are required (Wickens et al., 2010). These findings accord with prior research (Hyun & Luck, 2007) showing that when participants must make a mental transformation of information, performance suffers, as well as the broader notion of frame of reference transformations leading to errors (Wickens et al., 2010). The idea that spatial transformations underlie geospatial SA on our task is consistent with the strong relationship we observed between measures of spatial ability (MRT and VVP scores) and geospatial SA (see Table 1). However, the combination of response threshold (confidence) and *information quality* effects resulting from spatial alignment of HMIs in our study implicates a more nuanced effect than simply reducing the number of transformations. Spatial alignment fundamentally enhanced information uptake from the display, independent of the number of transformation stages, and despite the total visible information being identical. This finding, which could not have been uncovered without applying a cognitive model to examine latent processing variables, suggests a nonlinear interaction between information-processing speed and response-stages that may not be accounted for by our current understanding of geospatial SA (Frame of Reference, Visual Field Compatibility etc.). Understanding this interaction will be critical for future system design.

In sum, there is substantial evidence that alignment between a display and environment reduces the burden of mental rotation on the operator, boosting geospatial SA. While we have provided this demonstration in the context of a simulated submarine operator role, our findings potentially have broader implications for other modern work contexts that involve remote representations of external environments. For example, the visual field compatibility principle (Worringham & Beringer, 1989) has typically been studied in tasks involving single narrow fields of view to move remote levers or dials (Chan & Hoffman, 2016; Hoffman & Chan, 2013). Our findings should have relevance here, for example adding to the literature on exo- versus egocentric viewpoints for remote-controlled operation of UAVs and other vehicles (Higuchi & Rekimata, 2013). Our results could be used to make clear predictions for optimising displays and operator performance if system designers in those work contexts choose to employ wider fields of view, akin to the optronics 360° periscope display concept.

Although we acknowledge that pointing in the direction of contacts is not necessarily a major component of a periscope operator’s role in the submarine control room, it is worth noting that our results suggest that this task effectively tapped geospatial SA, which is likely to be important for periscope operators to complete their team task roles (see Michailovs et al., 2023; for example of team submarine control room experiment). In particular, the fact that geospatial SA correlated with general spatial ability supports the face validity of our experimental paradigm. That said, it is possible that our results may partially reflect a conflict between display location and response direction. The Simon effect describes an increment in RT for trials in which the stimulus is presented on the opposite side to the response, even when the decision to be made is a simple one (Hommel, 2011; Simon & Small, 1969). In the present task, a contact appearing on the right of the display but requiring a response to the left may be slower than one requiring a response to the right, even though the contact’s direction was determined just as quickly. However, in this paradigm that slowdown is, in effect, a reduced geospatial SA in the vein of Wickens et al. (2010) frame of reference transformations. In the context of emerging display technologies, our results show that geospatial SA decrements arising from configuration and display misalignment are an important consideration for submarine design and can be overcome with human machine interface (HMI) designs motivated by cognitive science.

It is also worth considering that HMIs that support geospatial SA could inhibit performance in other aspects of contact analysis—for instance, tracking moving contacts or assessing the distance between contacts. In this initial assessment of display configuration design, we used static, forward-facing contacts presented one at a time. In each of the panorama designs other than Clockwise, one or more strips was reversed to allow, for example, the Aft strip to show port on the left of screen. These spatial mapping discontinuities may make it more difficult to interpret a contact’s direction of travel, or the way strips connect to each other. Future research may consider using target contacts that move across the strips and ask participants to predict their future locations, or include multiple targets and asking participants to identify which are closest in bearing.

The Spatial configuration provided the greatest geospatial SA benefits, but it also represents the most substantial departure from the Clockwise panel design used by Michailovs et al., (2022, 2024). While all our configurations involved four display strips, for the Spatial configuration, these strips present eight sections of continuous horizon rather than the four such sections shown on other configurations. It remains to be seen whether such a design could incur deficits in other aspects of a periscope operator’s role. For instance, moving contacts would make frequent jumps across the smaller sections of the Spatial design versus a more traditional Clockwise configuration, and this additional transience may impact performance. This would represent an important extension of the current study. However, even more minor changes such as altering the top and bottom strips to display what is in front of and behind the operator (i.e. Partitioned design), respectively, provided geospatial SA benefits. Similarly, operators no longer need to be tethered to the periscope, allowing them to be positioned elsewhere in the submarine control room. We have found that the operator’s orientation does not impact their geospatial SA, but their orientation relative to the displayed content is a critical consideration in HMI design. This effect was less apparent in the Clockwise condition, which suggests that the Clockwise layout may allow a more flexible control room layout (but at the cost of overall operator geospatial SA). Alternative design concepts, such as the use of virtual reality head mounted displays should also be considered for their ability to create continuous 360° display options whilst preserving spatial relationships between the operator and Ownship.

### Conclusions

In summary, digital periscope technology provides a range of possibilities previously prohibited by a hull-penetrating optical path periscope, including the freedom to be oriented in any direction and the ability to view the whole horizon on a single display. Michailovs et al. (2022, 2024) have already shown benefits to a panoramic HMI design, as compared to a single-bearing view. The present study builds on this by showing that operator performance can be further facilitated by designs that maximise the alignment of the operator’s display to the external world, thus reducing the need for mental transformations to determine contact location. 

## Figures and Tables

**Fig. 10:**
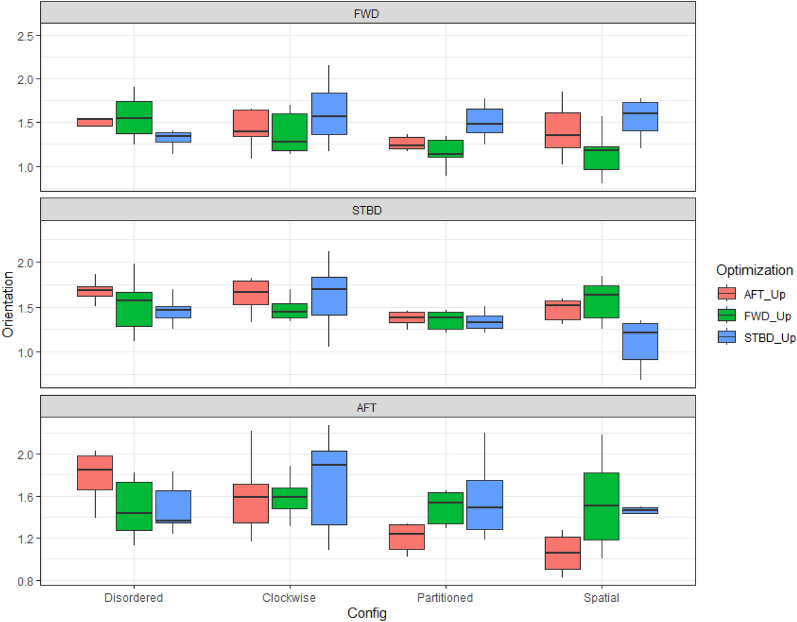
Mean response time (for correct responses) split by the between-subject factors display configuration (*x*-axis) and Optimisation (see legend), and the within-subject factor operator orientation (panels below each grey subheading)

**Fig. 11:**
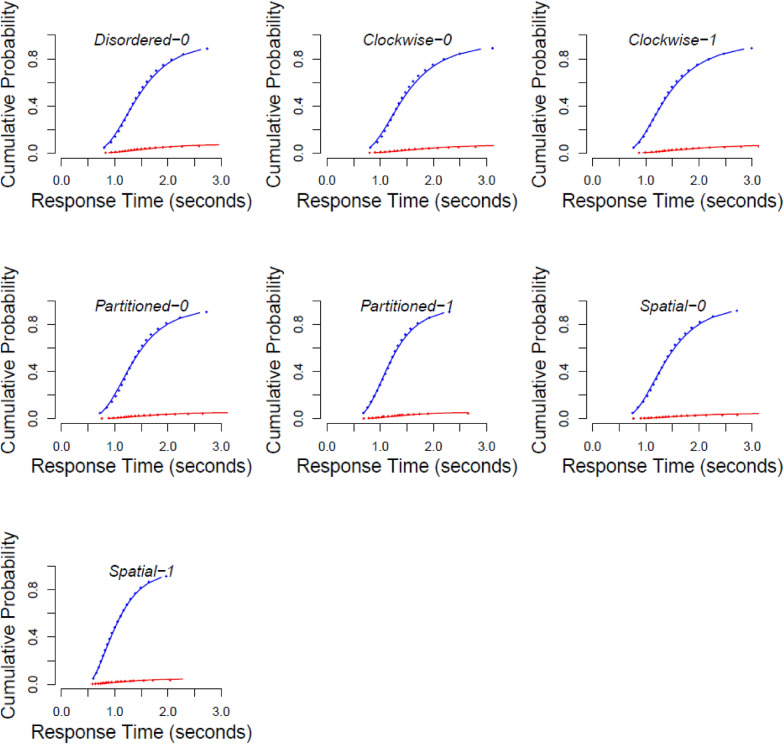
RDM quantile fits. The blue and red filled circles represent the 5th to 95th quantiles (in increments of 5%) of correct and error RTs, respectively, while the lines reflect RTs simulated from the group-level model parameters. Conditions are defined by the strip configuration, and Aligned (1) versus Misaligned (0)

## Data Availability

The datasets used and/or analysed during the current study are available from the corresponding author on reasonable request.

## Appendix

### Analysis of mean RT data by condition

Our experimental design led to a three-factor analysis, with Configuration (Disordered, Clockwise, Partitioned, Spatial) x Orientation (AFT, FWD, STBD) x Display-Optimised (AFT, FWD, STBD). In a Bayesian Three-way analysis of variance (ANOVA), the preferred model included all main effects and interactions (posterior probability > 0.99). While the results appear complex in Fig. 10, by far the strongest evidence was for the Orientation x Optimisation (BF_10_ > 1000) and the three-way (BF_10_ > 1000) interactions. When adjusting the other effects against matched models there was little-to-no evidence for the importance of other factors. Based on these analyses and interpreting the data in Fig. 10, it is clear that, as predicted, the best geospatial SA (fastest RT) depends not only on the display configuration, but how that configuration is optimised with respect to the orientation of the operator. For instance, consider the Spatial configuration condition (final three boxes in each panel of Fig. 10), which we predicted to produce best performance but only if the display configuration is optimised for the operator’s orientation. The data fit this prediction, i.e. when an operator is facing forward (FWD) RTs are the fastest overall when the Spatial configuration is matched to the operator orientation (FWD UP [green]) but substantially slower if the same configuration is not optimised/rotated for their position (i.e. slower for FWD facing but AFT-up [red] or STBD-up [blue]). This optimisation shifts accordingly for starboard (middle panel) and aft (lower panel) facing operators. These trends are less clear in the more partially aligned designs (Partitioned and Clockwise). The pattern of data then are best understood in terms of being Aligned or Misaligned, as we report in Fig. 6 of this study (Table

**Table 4:** Median RT and mean accuracy (95% CIs) by Config x Optimisation x Orientation

Config	ConfigOptimised	Orientation	MedianRT	CI_low_RT	CI_high_RT	Accuracy	CI_low_Acc	CI_high_Acc
Clockwise	AFT_Up	AFT	1.412	1.378	1.446	0.936	0.924	0.947
Clockwise	AFT_Up	FWD	1.346	1.310	1.382	0.941	0.930	0.952
Clockwise	AFT_Up	STBD	1.493	1.454	1.532	0.865	0.850	0.881
Clockwise	FWD_Up	AFT	1.437	1.403	1.471	0.926	0.913	0.939
Clockwise	FWD_Up	FWD	1.204	1.173	1.235	0.958	0.948	0.968
Clockwise	FWD_Up	STBD	1.341	1.310	1.372	0.952	0.941	0.963
Clockwise	STBD_Up	AFT	1.435	1.394	1.477	0.899	0.886	0.913
Clockwise	STBD_Up	FWD	1.434	1.400	1.468	0.915	0.902	0.928
Clockwise	STBD_Up	STBD	1.491	1.452	1.529	0.935	0.924	0.947
Disordered	AFT_Up	AFT	1.635	1.596	1.674	0.940	0.928	0.952
Disordered	AFT_Up	FWD	1.406	1.375	1.436	0.956	0.946	0.966
Disordered	AFT_Up	STBD	1.592	1.561	1.623	0.967	0.958	0.976
Disordered	FWD_Up	AFT	1.346	1.314	1.378	0.951	0.940	0.962
Disordered	FWD_Up	FWD	1.432	1.400	1.464	0.928	0.916	0.941
Disordered	FWD_Up	STBD	1.366	1.332	1.400	0.924	0.910	0.937
Disordered	STBD_Up	AFT	1.312	1.280	1.344	0.886	0.871	0.901
Disordered	STBD_Up	FWD	1.247	1.222	1.272	0.920	0.907	0.932
Disordered	STBD_Up	STBD	1.335	1.307	1.363	0.899	0.886	0.913
Partitioned	AFT_Up	AFT	1.184	1.155	1.213	0.962	0.952	0.971
Partitioned	AFT_Up	FWD	1.245	1.214	1.277	0.956	0.945	0.966
Partitioned	AFT_Up	STBD	1.345	1.310	1.380	0.947	0.935	0.958
Partitioned	FWD_Up	AFT	1.362	1.326	1.399	0.956	0.945	0.966
Partitioned	FWD_Up	FWD	1.132	1.103	1.161	0.973	0.965	0.981
Partitioned	FWD_Up	STBD	1.279	1.249	1.308	0.963	0.953	0.972
Partitioned	STBD_Up	AFT	1.369	1.334	1.404	0.929	0.917	0.941
Partitioned	STBD_Up	FWD	1.394	1.365	1.422	0.906	0.892	0.920
Partitioned	STBD_Up	STBD	1.231	1.205	1.258	0.953	0.943	0.963
Spatial	AFT_Up	AFT	0.946	0.925	0.967	0.968	0.959	0.977
Spatial	AFT_Up	FWD	1.296	1.266	1.327	0.964	0.955	0.973
Spatial	AFT_Up	STBD	1.316	1.287	1.345	0.951	0.940	0.962
Spatial	FWD_Up	AFT	1.351	1.313	1.389	0.951	0.941	0.962
Spatial	FWD_Up	FWD	1.028	1.002	1.054	0.977	0.970	0.985
Spatial	FWD_Up	STBD	1.386	1.348	1.424	0.943	0.931	0.955
Spatial	STBD_Up	AFT	1.380	1.350	1.409	0.970	0.962	0.979
Spatial	STBD_Up	FWD	1.414	1.383	1.445	0.955	0.944	0.965
Spatial	STBD_Up	STBD	1.017	0.991	1.042	0.963	0.954	0.973

^4^).

### Model fits

Below we present the group-level model fits for the RDM interpreted in the paper. The blue and red filled circles represent the 5th to 95th quantiles (in increments of 5%) of correct and error RTs, respectively, while the lines reflect RTs simulated from the group-level model parameters. In all but the Disordered condition, there is both a Misaligned (“0”) and an Aligned (“1”) within-subjects manipulation. These figures demonstrate that overall the model captured the trends of the data well, including both RT and accuracy. There are minor misfits, particularly in the extreme quantiles, which are to be expected (Fig. 11).

### Comparison of strip layout optimisation by operator orientation

In the following displays, we systematically demonstrate how each strip, for each condition x alignment, maps to the operator’s orientation.

## Abbreviations

AFT: Aft
BF: Bayes factor
EAM: Evidence accumulation model
FWD: Forward
GLMM: Generalized linear mixed-effects model
HMI: Human–machine interface
MRT: Mental rotations test
RDM: Racing diffusion model
RT: Response time
SA: Situational awareness
STBD: Starboard
VVP: Visualization viewpoints test
